# Precession and nutation dynamics of nonlinearly coupled non-coaxial three-dimensional matter wave vortices

**DOI:** 10.1038/srep22758

**Published:** 2016-03-11

**Authors:** R. Driben, V. V. Konotop, T. Meier

**Affiliations:** 1 Department of Physics and CeOPP, University of Paderborn, Warburger Str. 100, D-33098 Paderborn, Germany; 2 ITMO University, 49 Kronverskii Ave., St. Petersburg 197101, Russian Federation; 3 Centro de Física Teórica e Computacional and Departamento de Física, Faculdade de Ciências, Universidade de Lisboa, Campo Grande, Edifício C8, Lisboa 1749-016, Portugal

## Abstract

Nonlinearity is the driving force for numerous important effects in nature typically showing transitions between different regimes, regular, chaotic or catastrophic behavior. Localized nonlinear modes have been the focus of intense research in areas such as fluid and gas dynamics, photonics, atomic and solid state physics etc. Due to the richness of the behavior of nonlinear systems and due to the severe numerical demands of accurate three-dimensional (3D) numerical simulations presently only little knowledge is available on the dynamics of complex nonlinear modes in 3D. Here, we investigate the dynamics of 3D non-coaxial matter wave vortices that are trapped in a parabolic potential and interact via a repulsive nonlinearity. Our numerical simulations demonstrate the existence of an unexpected and fascinating nonlinear regime that starts immediately when the nonlinearity is switched-on and is characterized by a smooth dynamics representing torque-free precession with nutations. The reported motion is proven to be robust regarding various effects such as the number of particles, dissipation and trap deformations and thus should be observable in suitably designed experiments. Since our theoretical approach, i.e., coupled nonlinear Schrödinger equations, is quite generic, we expect that the obtained novel dynamical behavior should also exist in other nonlinear systems.

The discovery, theoretical investigation and experimental realization of novel nonlinear modes is a challenging quest in various research fields where nonlinear interactions play a vital role, such as photonics^1^, Bose-Einstein condensates (BECs)^2^, gas and fluid dynamics^3^ and several others. A special role among localized nonlinear modes play self-trapped modes, known as solitons^4^,^5^,^6^. Multidimensional solitary modes supported by a cubic self-focusing nonlinearity are, however, subject to collapse^5^,^6^,^7^. Also more complex multidimensional localized states carrying angular momenta in the form of vortex tori are vulnerable to azimuthal instability^8^,^9^,^10^, which splits them into fragments that later collapse.

Many schemes have been proposed to overcome the collapse and to stabilize multidimensional solitons by introducing linear or nonlinear stabilizing potentials^11^,^12^, by exploiting other than conventional Kerr type nonlinearities^13^,^14^,^15^ and by temporal modulations of the nonlinearity^16^,^17^. On the other hand fields under the action of a self-defocusing nonlinearity, trapped in localizing potentials such as the most popular parabolic one are able to support a remarkable variety of collapse-free modes without the application of sophisticated stabilization schemes. Some of these nonlinear modes of this model qualitatively overlap with their linear counterparts such as the harmonics of 3D quantum oscillator model. However, a self-defocusing nonlinearity may also support completely novel type of modes, that do not exist in the linear regime. A natural way to create these novel modes is by nonlinear coupling of the underlying linear modes.

The rich field of matter waves provides a physically realizable setting for our analysis. We chose here to work with 3D vortices that unlike fundamental localized ball-shaped modes provide an excellent possibility to exploit three dimensionality by the 3D choice of orientation of their axes. Working with 3D vortices is a natural choice for the present investigation, since the appearance of these object is ubiquitous in nature ranging from tornados to bubbles in liquids and gases emitted by cannons, volcanos and even by living organisms like dolphins.

In a 3D one-component BEC, where a vortex is characterized by its charge, geometry and vorticity line, vortices have received significant attention^2^,^18^. Multiple studies were devoted to vortex shapes and their stability^19^,^20^,^21^,^22^,^23^,^24^,^25^,^26^. Previous investigations of BECs concentrated on the dynamics of vortex lines, in particular Kelvin waves^19^,^27^, vortex clusters^28^, and multipoles^29^,^30^,^31^, as well as on the existence of more complex topological objects like hybrid vortex solitons^32^ and twisted 3D vortex solitons^33^ in inhomogeneous media. Even richer topological structures appear in multi-component condensates^34^. These are, in particular, mixtures of hyperfine states, like the ones explored since the very first experiments that reported the observation of vortices in BECs^35^,^36^,^37^,^38^. Such multi-component BECs have a spinor nature and are characterized by macroscopic order parameters with two or more components. Also the complex dynamics of phase separation^39^ and sophisticated topological states like vortex-soliton pairs^40^, skyrmions^41^,^42^, vortex lattices^43^, and Dirac magnetic monopoles^44^ have been described theoretically and observed experimentally in spinor BECs.

Here, we analyse and predict results of the unconventional dynamics of nonlinearly coupled vortices whose lines are orthogonal each other. We call them “cross-vortices”. These objects create families of solutions that are parametrized by the chemical potential (or by a number of atoms) and that emerge from the linear states due to the nonlinear inter-species interactions. The inter-species interactions depend on the angle between the vortex lines and achieve minima exactly when the vortex lines are orthogonal each other. The cross-vortices are observed in miscible configurations where each component has a nonzero density both inside and outside the core region of the other component and is characterized by a hole where the vortex lines intersect. In the presence of interactions these objects support several persistent dynamical regimes when excited from the linear modes having a mutual relative orientation represented by arbitrary relative angles. Unlike the BEC gyroscopes suggested in^19^ whose dynamics display precession of the vortex lines, we show here persistent precession accompanied by nutations, which closely resemble the dynamics of a heavy top^45^. The dynamics reported here is based on careful direct numerical tracking of vectors of angular momenta of the interacting 3D vortices that is combined with a theoretical analysis. We also demonstrate that is should be possible to realize the discovered dynamics experimentally in BECs including imperfections such as dissipative effects and traps with a non-perfect symmetry. The predicted novel dynamics with distinct features of both wave and rigid body mechanics are a manifestation of a pure nonlinear phenomenon as the inter-species interactions create the force that induces the nutations of the vortex lines.

## Results

### The model and stationary orthogonal 3D vortices

We consider a binary mixture of BECs of the atomic components having equal atomic masses *m* and loaded in radially-symmetric identical parabolic traps characterized by the linear oscillator frequency *ω*_0_. We also assume that the intra-species scattering lengths *a*_1,2_ are equal, i.e., *a*_1_ = *a*_2_ = *a*_*s*_, and the inter-species interactions are characterized by the s-wave scattering length *a*_12_. Further, we adopt dimensionless units where time and coordinates are measured in 2/*ω*_*ho*_ and 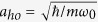 units, respectively. The mixture is described by the two-component macroscopic wave function (spinor) Ψ = (*ψ*_1_, *ψ*_2_)^*T*^ which solves coupled dimensionless Gross-Pitaevskii equations (GPEs)


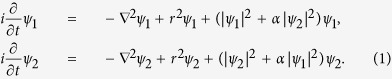


Here, *α* = *a*_12_/*a*_*s*_ ∈ [0, 1] characterizes the relative strength of the inter- and intra-species interactions; we consider positive scattering lengths when the homogeneous mixture is miscible (a stratified phase is absent^46^). The total number of atoms is given by N= *Na*_*ho*_/(8*πa*_*s*_) with 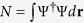. Equations (1) conserve the number of atoms in each component, i.e., *N*_1,2_, the total energy *E* = *E*_1_ + *E*_2_ + *E*_*int*_ where 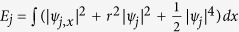 is the energy of the *j*-th component and 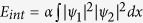 is the energy of inter-species interactions, as well as the total angular momentum **L** = **L**_1_ + **L**_2_ where 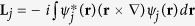. Neither the energies *E*_*j*_ nor the angular momenta **L**_*j*_ are conserved, but


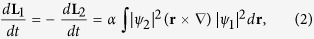


i.e., the individual angular momenta change because of the inter-species interactions. We also notice that in the model (1) intraspecies interactions are chosen equal. This, however is not a limitation for the observed modes and their dynamics, the later being structurally stable. Below we show the structural stability of the observed effect, which can be observed for non-equal values of the scattering lengths, in particular for condensates of rubidum atoms (see Eq. (5) and Fig. 5 below).

Usually, the generation of single vortices is achieved either by rotating traps^18^ or by dynamical^35^ and topological^38^,^47^ phase imprinting. Due to the topology, i.e. because of the orthogonality of the vorticity lines, the above techniques appear to be not suitable for the generation of cross-vortices. Therefore, we adopt for our model (1) the following methodology. Initially two (linear) vortices are created in the absence of two-body interactions and thus independently in each of the components. Then the nonlinearity is switched on, which can be realized by means of the Feshbach resonance. Even though this represents a rather strong initial “perturbation”, we find that the objects created in such a way are robust cross vortices, which are accompanied by rotational dynamics. We notice that previously the vortex states continued from the linear limit were discussed in the literature^21^, however, in the context of a one-component condensate.

The described approach has two additional features relevant to our case. First, by exciting a pure linear mode one can easily impose the required topology, i.e. create desirable vorticies of the components. Second, the cross-vortices considered here have a linear limit and are reduced to the harmonic oscillators eigenmodes in the limit of small number of atoms. This is particularly clear from the analysis of the stationary solutions of (1) with components having equal chemical potentials: *ψ*_*j*_ = *e*^−*iμt*^*u*_*j*_(**r**), where *u*_*j*_ solve the stationary GPEs (*j* = 1, 2)


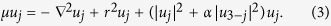


In the linear case 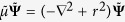 (tildes denotes the linear eigenvalues and -functions) the components are decoupled and the eigenstates are the linear harmonic oscillator eigenmodes. We fix the *z*-axis along the vortex line of the simplest linear vortex of the first component, i.e., 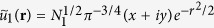, which corresponds to 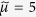 and has *N*_1_ atoms (changing the vortex line direction along its axis affects only the direction of the rotations). The vortex line in the second component is taken to be rotated by an angle *β* with respect to the *y*-axis, i.e., 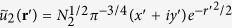, with


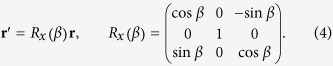


**Figure 1 f1:**
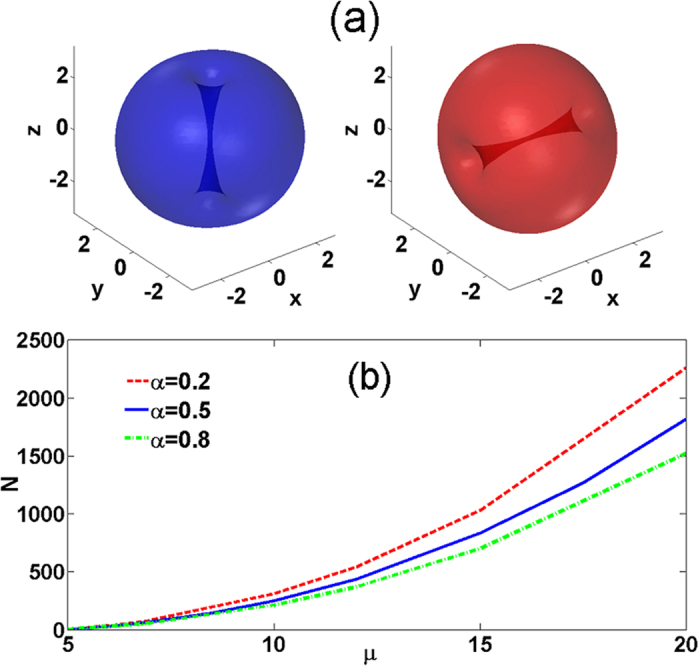
(**a**) Isosurfaces of the components of the cross vortex corresponding to  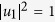 and  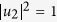 for *μ* = 10. (**b**) Dependence *N vs μ* for several values of the interspecies interactions.

Considering a family of nonlinear solutions with the origin at a (linearly degenerate) state of the spinor 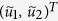, the interaction energy deposited into the system when a weak nonlinearity is switched-on is 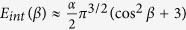. *E*_*int*_(*β*) achieves its minimum at *β* = *π*/2 when the two vortex lines are orthogonal, see Fig. 1(a). A family *N*(*μ*) of nonlinear cross–vortices has its origin at the state 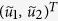 corresponding to *β* = *π*/2. The isotropy of the system with respect to the *y* axis suggests that the entire family, i.e., the nonlinear cross vortices, are also characterized by orthogonal vortex lines. Such families exist for several values of the interspecies interaction *α*, see Fig. 1(b). It is evident that all these families grow from a single solution corresponding to 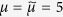. For simplicity we adopt in what follows the value of *α* = 0.5.

### Persistent dynamics of precession with nutations

**Figure 2 f2:**
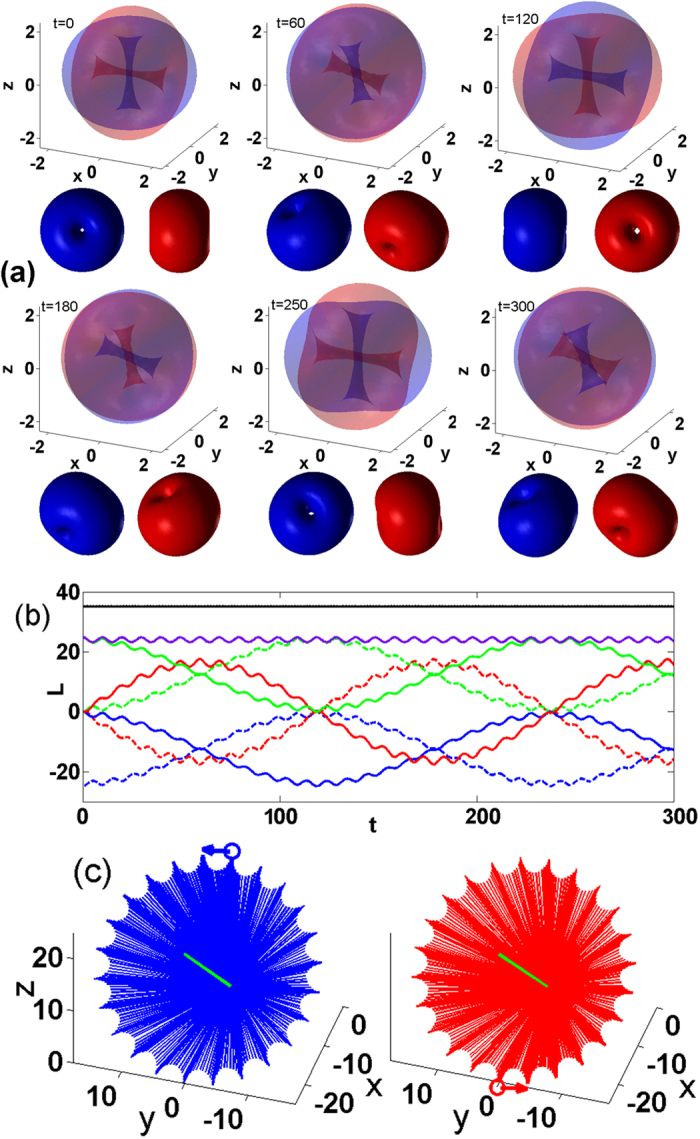
(**a**) Snapshots of the cross vortex dynamics with N = 25 at times indicated in the panels. The isosurfaces correspond to 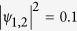. Blue and red colors represent the *ψ*_1_ and *ψ*_2_ components, respectively. (**b**) The solid (dashed) lines show the dynamics of the *x* (blue), *y* (red), and *z* (green) components of the angular momentum **L**_1_ (**L**_2_). The moduli of **L**_1_ and **L**_2_ are shown by the overlapping purple lines and the conserved modulus of the total angular momentum **L** by the solid black line. (**c**) The evolution of **L**_1_ (left panel) and **L**_2_ (right panel). The single green lines represent the invariant **L** and circles with arrows the axes positions at *t* = 0.

Here we analyze dynamics of our system by computing the evolution of a cross vortex after an instantaneous switch-on of the nonlinearity. We therefore solve (1) starting from initial conditions 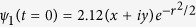, 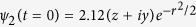 with *N*_1_ = *N*_2_ ≈ 25. Snapshots of the evolution of the binary condensate are shown in Fig. 2(a). We observe that both vortex lines experience persistent rotations returning to the initial state after the full period *T* ≈ 248. For a typical linear trap frequency of 80 Hz the precession period is approximately 1 s which allows the observation of several precession periods in experimentally available condensates.

**Figure 3 f3:**
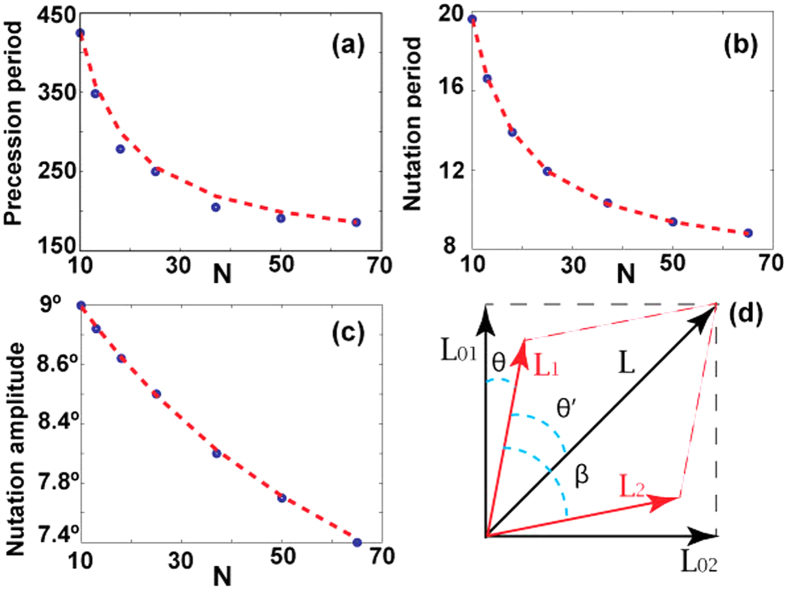
(**a**) Computed precession periods (circles) and interpolated by 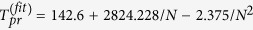 (dashed line). (**b**) Computed nutation periods (circles) and interpolated by 
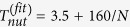
 (dashed). (**c**) Computed nutation amplitude (circles) and interpolated by *θ*^(*fit*)^ = 9.5 + 0.056 *N* − 5.2 · 10^−4^ *N*^2^ − 2.3 · 10^−6^ *N*^3^ (dashed). (**d**) Schematic diagram of the angular momenta.

In Fig. 2(b) we present the evolution of the projections of **L**_1,2_. The dynamics of the angular momentum components shows two types of oscillations. Oscillations with a longer period and larger amplitude correspond to rotation of the individual momenta **L**_1,2_ with respect to the conserved **L**, i.e., the *precession* of the cross vortex. The precession is accompanied by oscillations with a smaller period and amplitude. The amplitude of these oscillations is characterized by the angle *θ* measuring deviations of **L**_1,2_ from their initial orientations **L**_*j*_(*t* = 0) = **L**_0*j*_ [see Fig. 3(d) below] corresponding to *nutation*. Figure 2(c) shows a vectorial representation of the evolution of the components’ angular momenta **L**_1,2_.

Our simulations demonstrate the robustness of the nonlinear cross vortices, even though they are excited by initial conditions that significantly deviate from stationary nonlinear vortices. The dynamics is accompanied by modulations of the vortex lines and of the vortex shapes. Numerical studies performed for different *N* result in qualitatively similar regimes, however, the dynamical characteristics are significantly affected by the two-body interactions. This is illustrated in Fig. 3 where we show the precession [Fig. 3(a)] and nutation [Fig. 3(b)] periods and the nutation amplitude [Fig. 3(c)] as function of the number of particles. For small *N* the precession and nutation are slow and they accelerate as *N* increases, while the nutation amplitude decreases.

**Figure 4 f4:**
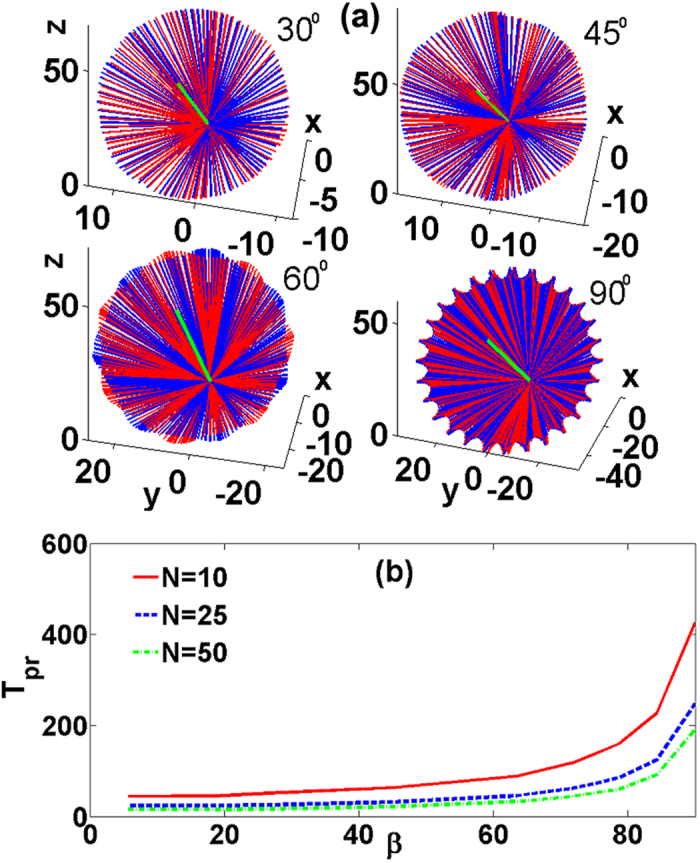
(**a**) Precession of nonlinear vortices grown from the linear harmonics with *N* = 50.11 and various values of *β*. The axes of *ψ*_1_ (blue) and *ψ*_2_ (red) are merged into single panels. (**b**) Dependence of the precession period on the initial angle *β*(*t* = 0).

The observed dynamics is not obvious for at least two reasons. First, earlier reported precession of vortices was explained by instabilities^22^,^23^. We, however, did not observe a transient period corresponding to development of instabilities but the rotational dynamics started immediately after switching-on the nonlinearity. On the other hand, Eq. (2) indicates that a “force” necessary to induce precession and nutation should be related to anisotropy of the atomic distributions in each of the vortices and cannot be induced at *t* = 0 by the imposed initial conditions, i.e., the right hand side of Eq. (2) is zero for any vortex of the form 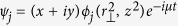, where **r**_⊥_ = (*x*, *y*). To shine light on the origin of the observed evolution we use a simple mechanical analogy shown in Fig. 3(d). The initial angular momenta **L**_01_ and **L**_02_ of the linear modes, can be viewed as an initial perturbation of the angular momenta of the nonlinear cross-vortex with **L**_1_ and **L**_2_. Since **L**_1_ · **L**_2_ ≠ 0, i.e., *β*(*t* = 0) < *π*/2, the dynamics show a precession whose period is faster for a smaller *β*(*t* = 0) [see Fig. 4(b)]. Concomitantly, due to the inter-atomic interactions there appears a “force” tempting to restore *β* = *π*/2 [i.e. to minimize *E*_*int*_(*β*)]. This force is responsible for the nutation. We also verify that 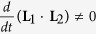 (notice that the components of **L**_1_ and **L**_2_ oscillate out-of-phase), i.e., the moduli *L*_1,2_ are not constant. We have found that only configuration with *β* = *π*/2 gives rise to stationary nonlinear modes. However if in the input we have not these exact stationary nonlinear modes, but perfectly orthogonal vortices corresponding to the linear case we observe persistent dynamical modes of double precession with nutations by instantaneous switching on the nonlinearity. For the configurations of *β* different from 0.5 *π* we can induce only persistent dynamical modes from linear vortices by instantaneous switching of the nonlinearity. Thus *β* < *π*/2 influences an “initial” angular velocity of the precession (*β* = *π*/2 corresponds to zero velocity). This difference in the initial angular velocities is clearly visible when comparing the dynamics shown in Fig. 3 for different initial angles.

Since both precession and nutation originate from the nonlinear interspecies interaction they disappear in the linear limit (*N* → 0), i.e., the periods become infinite. Thus 1/*N* can be viewed as a small parameter for the estimate of the periods. Figure 3(a,b) show the respective fitting curves. The dependence of the angular nutation amplitude is approximated by an almost linear dependence on *N*, see Fig. 3(c).

Although, the presented “mechanical” picture is over-simplified, it nevertheless results in reasonable estimates for the dynamical parameters. If 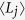 is the average value of  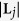, the average angle of precession can be estimated as 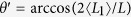 and the average angle of nutation as *θ* = *π*/4 − *θ*′, respectively. Thus the ratio between the nutation and precession periods can be estimated as *T*_*nut*_/*T*_*pr*_ = sin(*θ*′)/sin(*θ*). Using these simple estimates for the case of *N* = 25 shown in Fig. 3, we obtain *θ*′ ≈ 0.75196, *θ* ≈ 0.03344 and hence *T*_*nut*_/*T*_*pr*_ ≈ 20.42. The result of our simple considerations is not very far from the numerically observed value of (*T*_*nut*_/*T*_*pr*_)_*num*_ ≈ 25.05 but clearly deviates from it. This difference can be explained by the strength of the inter-atomic interaction which results in a significantly perturbed nonlinear dynamics. Reducing the number of particles to *N* = 10 (not shown here), i.e., reducing the nonlinearity, one obtains much “cleaner” precession and nutation. For this case, our estimate yields *θ*′ ≈ 0.7537, *θ* ≈ 0.0317 and hence *T*_*nut*_/*T*_*pr*_ ≈ 21.6 while numerically we find (*T*_*nut*_/*T*_*pr*_)_*num*_ ≈ 21.9 which is a remarkable accuracy.

A similar dynamics is also observed for arbitrary initial angles between **L**_1_ and **L**_2_. We start again from the linear 3D harmonics oriented this time arbitrary with respect to each other and instantaneously switch-on the nonlinear interaction. For examples of the vortex precession and nutation for different initial *β* see Fig. 4(a). The dynamics obtained for *β* < *π*/2 are clearly different from those for *β* = *π*/2 since for *β* ≠ *π*/2 instead of spikes smooth oscillations appear which correspond to a precession with a forward release speed^45^. For small *β* the amplitude of the nutations decreases significantly and is hardly observable. Also for smaller values of *β* the precession is much faster and consequently the opening angles of axes cones decrease with *β* as can be seen comparing the scales of panels of Fig. 4(a). Figure 4(b) shows the dependence of the full precession period on the initial angle *β*. For all considered *N* the period decreases strongly when *β* deviates from *π*/2. We have performed systematic studies of the persistent dynamical modes varying the XPM/SPM strength ratio. The results showed that qualitatively the dynamics remains robust with only qualitative dependence of the nutations and precession periods variations.

### Dynamics including dissipation and different interspecies interactions

Regarding possible sources of dissipative losses of atomic condensates we tested our model for inelastic inter-atomic interactions, inelastic interactions of atoms with the trap and for linear losses. These three effects all resulted in a gradual decrease of the number of atoms with a consequent decrease of the angular momenta. The dissipative models exhibit, however, all main features of the conservative case. In particular, the persistent joint dynamics of the two orthogonal vortices is completely preserved featuring rotation of angular momenta orientation accompanied by nutations. However, due to the gradual decrease of the densities, the strength of the nonlinear interaction between the vortices decrease and therefore the nutation periods increase and the total motion trajectory of vortices locus becomes a spiral instead of a perfect circle in conservative model.

An important point, is the effect of difference of intraspecies interactions of the two components. To illustrate the robustness of the all observed effects we now consider a practical model that is most closely related to experimental settings^48^, and which includes both non-equal interspecies interactions as well as inelastic inter-atomic interactions:


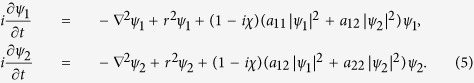


Here *a*_11_/*a*_12_/*a*_22_ = 100.4/98.006/95.44 corresponding to rubidium isotopes and the scattering lengths have small complex parts described by 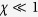. For the numerical simulations we consider *χ* = 0.005 which corresponds to the measured decay rate due to binary inelastic collisions^48^.

**Figure 5:**
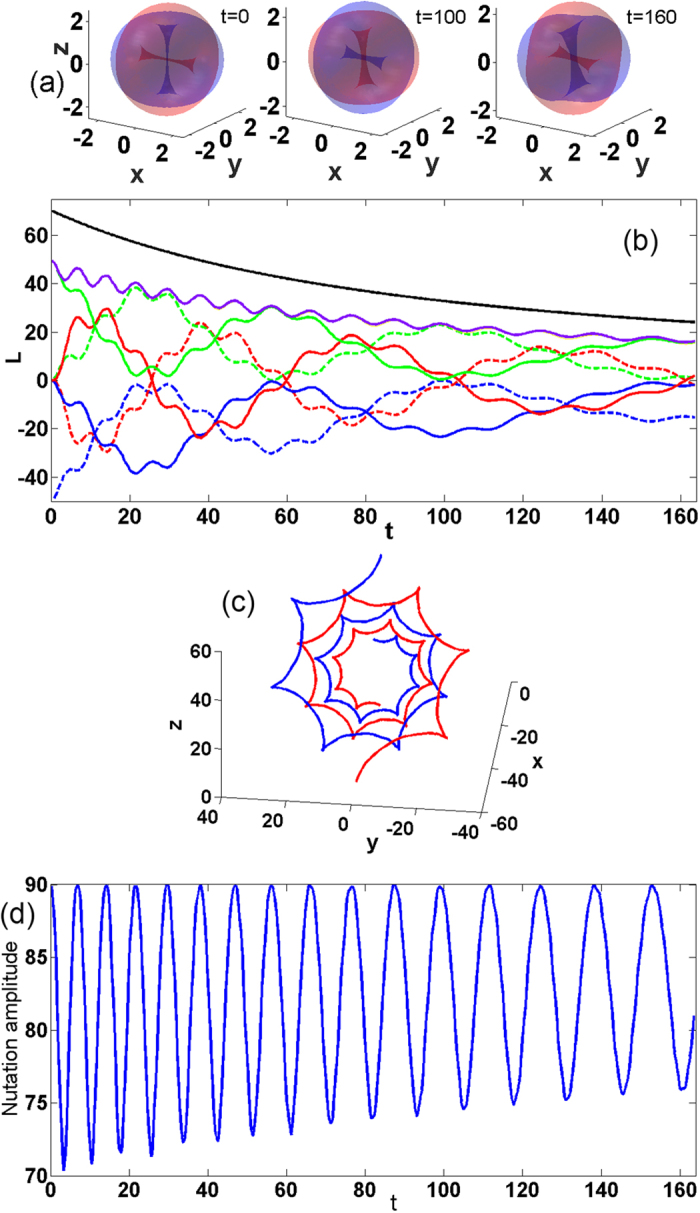
Dynamics of two orthogonal vortices in presence of dissipation. (**a**) Snapshots at three temporal points. The isosurfaces correspond to 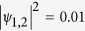. The widths of vortex lines at the snapshots clearly demonstrate the dissipation of the condensates. (**b**) Evolution of angular momenta in scalar representation (colors notations are as in Fig. 2(b) in the main text). (**c**) vectorial representation for angular momenta (colors notations are as in Fig. 2(c) in the main text). (**d**) Evolution of nutation amplitude with time. The dissipation parameter is *χ* = 0.005.

Figure 5 displays the evolution of the condensate for two full rotation periods in presence of the dissipation. Clearly, Fig. 5(a–c) show that the dissipation significantly reduces the norms of the two vortices and thus also reduces the total angular momentum. Therefore, the motion of the locus is a spiral instead of a closed circle. Figure 5(d) reveals that the nutations increase their periods while the amplitude of the nutation oscillations is decreasing.

### Dynamics in a non-spherical trap

**Figure 6:**
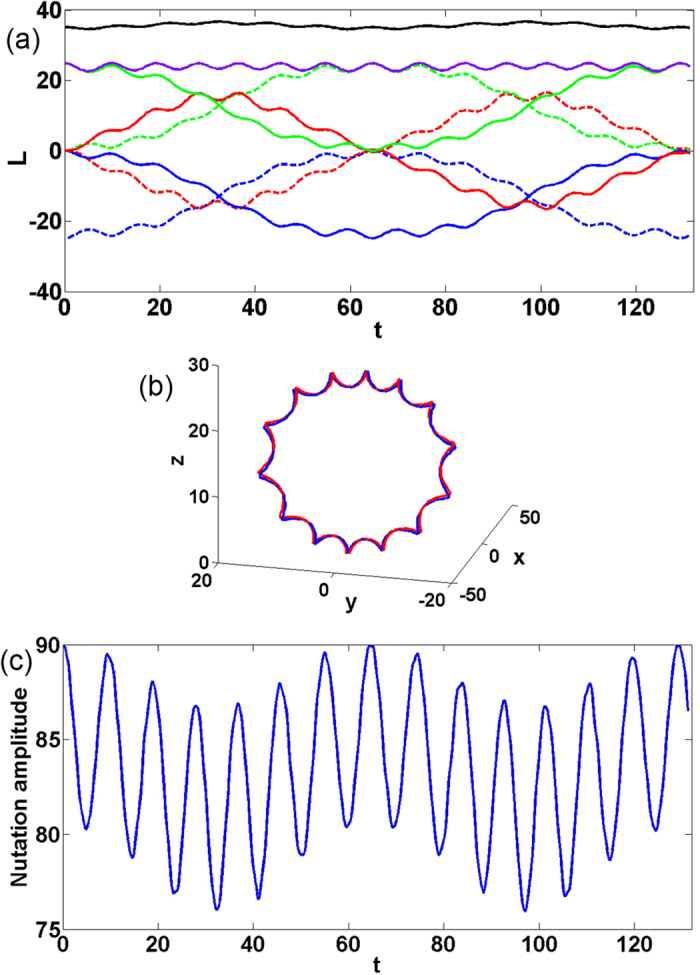
Dynamics of orthogonal vortices in case of the deformed trap with *v* = 1.02. (**a**) scalar representation (colors notations are as in Fig. 2(b)), (**b**) vectorial representation (colors notations are as in Fig. 2(c)), and (**c**) the evolution of the nutation amplitude with time.

Let us consider now the dynamics in a deformed trap, having an ellipsoidal form. Strong deviations from the spherical symmetry will destroy the persistent nature of the two condensate dynamics, however, if the deformations are not too strong the robust periodic oscillations of the two orthogonal vortices is preserved. To illustrate this, we analyze the case of deforming the trap in the direction orthogonal to the plane of the initial location of the vortex lines. More specifically, we consider a trap of the form


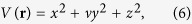


where *v* > 0 is the deformation parameter. Clearly, for *v* ≠ 1 the spherical symmetry is broken. Consequently the vector of the total angular momentum in a general case is not conserved any more. However, the isotropy of the potential with respect to *y*-axis holds, and thus the *y* component of the momentum is still conserved. In Fig. 6 we illustrate the obtained dynamics in a trap stretched in *y*-direction. We observe that the dynamics becomes accelerated, i.e., a faster exchange of angular momenta among the components and a about half the original total precession period. However, the main features of the precession and nutation dynamics is preserved in the non-sperical trap. As explained above, the combined angular momentum is not preserved (black curve in Fig. 6, the trajectory of motion of each vortex precession becomes prolate (see Fig. 6) and the amplitude of the nutation oscillations show a two-frequency beating due to the appearance of the additional frequency of the trap (see Fig. 6).

## Summary

In summary, the nonlinear interaction between two non-coaxial 3D vortices trapped in a parabolic potential has been analyzed and novel stationary and dynamically persistent modes are obtained. These robust dynamical modes can be excited from the noninteracting eigenstates of the 3D linear harmonic oscillator by sudden switch-on of the nonlinearity and they are characterized by remarkably persistent dynamical regimes of precession with nutation resembling the motion of a rigid body. Unlike in other cases, the smooth motion presented here starts immediately when the nonlinear interaction is present without any complex or chaotic intermediate regime. Whereas the torque-free precession is generated by the initial conditions, the nutation occurs due to the inter-species interactions and is associated with deformations of the vortex shapes. The reported effects are experimentally feasible because they are robust with respect to several effects including inelastic interactions with the trap and between the atoms^48^,^49^, change of the number of atoms and the nonlinear coefficients and weak non-spherical and anharmonic deformations of the trap. Our studies thus pave a way towards the manipulation and control of multidimensional matter wave vortices carrying angular momenta with the possibility of careful tracking and designing the trajectories of motion. Furthermore, we expect that the dynamical regime predicted here should also exist in other nonlinear systems.

## Additional Information

**How to cite this article**: Driben, R. *et al.* Precession and nutation dynamics of nonlinearly coupled non-coaxial three-dimensional matter wave vortices. *Sci. Rep.* **6**, 22758; doi: 10.1038/srep22758 (2016).
